# Chromosome substitution strain assessment of a Huntington’s disease modifier locus

**DOI:** 10.1007/s00335-014-9552-9

**Published:** 2015-02-03

**Authors:** Eliana Marisa Ramos, Marina Kovalenko, Jolene R. Guide, Jason St. Claire, Tammy Gillis, Jayalakshmi S. Mysore, Jorge Sequeiros, Vanessa C. Wheeler, Isabel Alonso, Marcy E. MacDonald

**Affiliations:** 1 Center for Human Genetic Research, Massachusetts General Hospital, Boston, MA 02114 USA; 2 UnIGENe, Institute for Molecular and Cell Biology (IBMC), University of Porto, 4150-180 Porto, Portugal; 3 CGPP, Institute for Molecular and Cell Biology (IBMC), University of Porto, 4150-180 Porto, Portugal; 4 Instituto de Ciências Biomédicas Abel Salazar (ICBAS), University of Porto, 4050-313 Porto, Portugal; 5 Center for Human Genetic Research, Massachusetts General Hospital, Simches Research Building, Room 5414, 185 Cambridge Street, Boston, MA 02114 USA

## Abstract

Huntington’s disease (HD) is a dominant neurodegenerative disorder that is due to expansion of an unstable *HTT* CAG repeat for which genome-wide genetic scans are now revealing chromosome regions that contain disease-modifying genes. We have explored a novel human–mouse cross-species functional prioritisation approach, by evaluating the HD modifier 6q23–24 linkage interval. This unbiased strategy employs C57BL/6J (B6J) Hdh^Q111^ knock-in mice, replicates of the HD mutation, and the C57BL/6J-chr10^A/J^/NaJ chromosome substitution strain (CSS10), in which only chromosome 10 (chr10), in synteny with the human 6q23–24 region, is derived from the A/J (AJ) strain. Crosses were performed to assess the possibility of dominantly acting chr10 AJ-B6J variants of strong effect that may modulate CAG-dependent Hdh^Q111/+^ phenotypes. Testing of F1 progeny confirmed that a single AJ chromosome had a significant effect on the rate of body weight gain and in Hdh^Q111^ mice the AJ chromosome was associated subtle alterations in somatic CAG instability in the liver and the formation of intra-nuclear inclusions, as well as DARPP-32 levels, in the striatum. These findings in relatively small cohorts are suggestive of dominant chr10 AJ-B6 variants that may modify effects of the CAG expansion, and encourage a larger study with CSS10 and sub-strains. This cross-species approach may therefore be suited to functional in vivo prioritisation of genomic regions harbouring genes that can modify the early effects of the HD mutation.

**Electronic supplementary material:**

The online version of this article (doi:10.1007/s00335-014-9552-9) contains supplementary material, which is available to authorized users.

## Introduction

Huntington’s disease (HD) is a dominantly inherited neurodegenerative disorder, usually of adult onset, characterised by involuntary choreic movements, cognitive impairment and behavioural changes. It is caused by the expansion of an unstable polymorphic CAG repeat in *HTT* (previously *HD*) (The Huntington’s Disease Collaborative Research Group 1993). The expanded CAG repeat is the major determinant of age at onset of motor symptoms, such that the longer the repeat the earlier the onset. It explains about 50–70 % of the variance in motor age at onset (Andrew et al. 1993; Duyao et al. 1993; Snell et al. 1993), while the remainder of the variance is highly heritable strongly implying the existence of genetic factors that modulate the rate of the pathogenic process that leads to onset of symptoms (Djousse et al. 2004; Gusella and MacDonald 2009; Wexler et al. 2004).

Amongst other potential modifier loci identified in genome wide linkage scans (Gayan et al. 2008), a scan using affected HD sibling pairs, the HD-MAPS study, has provided strong evidence for a quantitative trait locus (QTL) at the 6q23–24 modifying residual age of onset of neurological symptoms (Li et al. 2003, 2006). This 6q23–24 interval contains at least 80 genes, making it challenging to identify the gene (or genes) with the functional variant(s) that actually modify onset. Furthermore, unbiased linkage and association studies are gaining momentum and undoubtedly will identify many more genomic regions. These can be dissected with follow-up genetic and genomic studies with additional subjects and the recent advent of highly efficient gene targeting techniques now permit studies of candidate gene variants in human HD cells. However, efficacy in modulating the *HTT* CAG-initiated disease process and its outcomes in vivo may be needed to reveal modifier effects at each of many implicated modifier loci across the genome but is impractical on a gene-by-gene basis.

Therefore, we have explored an unbiased cross-species genetic approach to the problem of prioritising and homing in on HD modifier genes that utilises a mouse resource that has proven to be a powerful route to identification and validation of QTLs: the chromosome substitution mouse strains (CSSs). These mice, commonly referred as CSS*i,* carry both copies of a chromosome *i* from a donor strain, e.g. A/J (AJ), while all other chromosomes from the host strain, e.g. C57BL/6J (B6J), are intact and homozygous (Nadeau et al. 2000). By testing mice from a CSS strain for a phenotype of interest, it can be immediately inferred that phenotypic differences between the CSS and the host strain are due to at least one QTL resident on the substituted chromosome. The same principle can be used to identify regions carrying potential genetic modifiers whose effects may only be revealed in the presence of the primary mutation that is necessary to produce the phenotype that is modulated. The cross between CSSs mice and mice carrying a CAG expansion allele that precisely replicates the HD mutation, such as Hdh^Q111^ knock-in mice (Wheeler et al. 1999; White et al. 1997), could therefore provide an unbiased route to prioritise and validate in vivo the initially large genomic regions that harbour HD modifier loci. The Hdh^Q111^ knock-in mice exhibit accurate expression of mutant huntingtin from CAG repeats inserted into the mouse HD gene homologue (*Htt*, formally *Hdh*) and heterozygous mutant mice display phenotypes at a young age that are dominant, *Htt* CAG repeat length-dependent and manifest in medium spiny neurons (MSNs) (Wheeler et al. 2000, 2002), conforming to the key genetic features of the HD mutation. Theoretically, a difference in any of these phenotypes between the F1 progeny and parental knock-in mice implies that at least one or more genetic modifiers of HD mutation-initiated disease process reside on the substituted chromosome.

In this range-finding study, we have explored this approach, by applying it to the human 6q23–24 region implicated in harboring modifier(s) of HD onset. Since the human chromosome 6 is in synteny with the mouse chromosome 10 (chr10), we used a cross between CSS10 mice and B6/J Hdh^Q111/+^ knock-in mice to generate progeny with one copy of an AJ chr10 and heterozygous for the HD mutation in an otherwise B6J background. Comparing these F1 mice with B6/J Hdh^Q111/+^ knock-in mice tests if chr10 AJ-B6J genetic variants are of sufficient effect size to alter early, dominant and CAG length-dependent phenotypes, such as *Htt* CAG somatic instability (Lee et al. 2010, 2011; Wheeler et al. 1999), intranuclear inclusions of mutant huntingtin (Wheeler et al. 2002) and expression of dopamine- and cAMP-regulated phosphoprotein, 32 kDa (DARPP-32). Further motivating this comparison, Hdh^Q111^ and other *Htt* CAG knock-in mice exhibit decreased body weight, which on other genetic backgrounds appears to manifest at old ages as a low penetrant phenotype (Holter et al. 2013; Lin et al. 2001), while CSS10 mice carry chr10^A/J^ variants that moderate the rapid B6J weight gain associated with an A/J obesity-resistance QTL at chr10 (Burrage et al. 2010; Singer et al. 2004). We hypothesised, therefore, that rate of weight gain may provide an HD repeat relevant primary phenotype with which to calibrate our ability to detect the functional effects of chr10 variants in our test cross.

## Materials and methods

### Mice

**Fig. 1:**
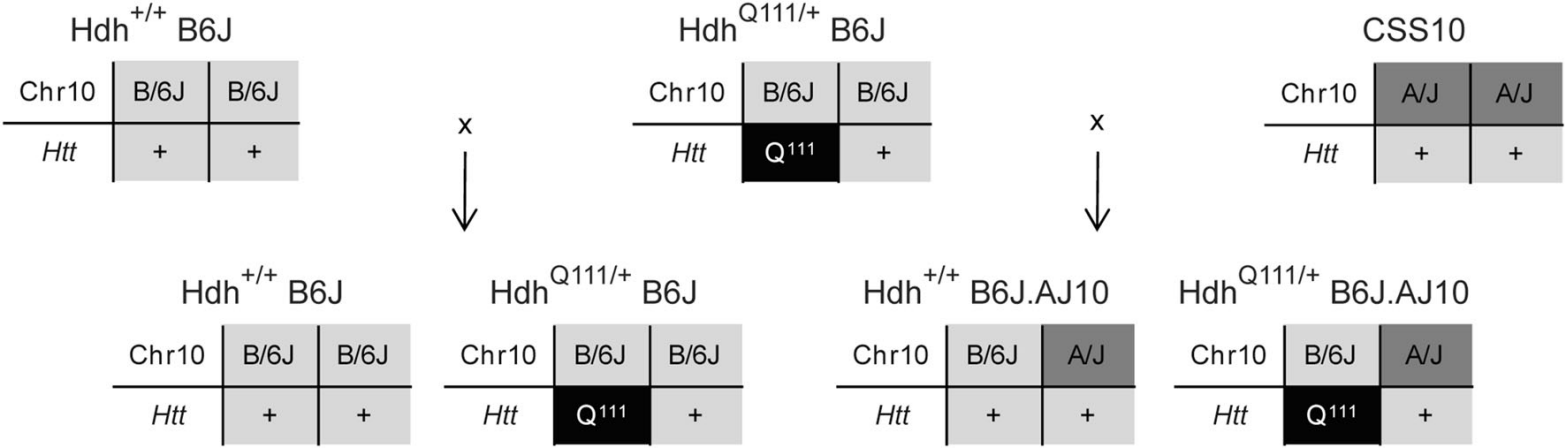
Hdh^Q111^ knock-in mice with different chr10 genetic backgrounds. Representative diagram of the breeding scheme showing the manner in which both chr10—AJ or B6J—and *Htt* alleles—wild type (+) or Hdh^Q111^ knock-in allele (Q^111^)—were passed to the F1 progeny mice used in this study. The actual CAG size of the Hdh^Q111^ knock-in allele in the paternal mice was 139 CAGs while in the F1 progeny ranged from 133 to 149 CAGs

The C57BL/6J-Chr10^A/J^/NaJ (CSS10) mice that carry both chr10 from strain AJ on an otherwise C57BL/6J (shortened here to B6J) background (Nadeau et al. 2000) and wild type B6J mice were obtained from The Jackson Laboratories (Jackson Laboratories, Bar Harbor, ME). The congenic Hdh^Q111^ knock-in line of mice used in this study carry the targeted *Htt* CAG expansion allele (Wheeler et al. 1999; White et al. 1997) on the B6J background. The renaming of the human HD gene, from *HD* to *HTT*, and of its murine homologue, from *Hdh* to *Htt*, has obscured connexions to previous research. Therefore, to maintain consistency with the literature and with the names of the lines of mice deposited in repositories, we use Hdh^Q111^ to denote the published name of the specific line of mice that we have utilised. The locus name *Htt* is used to refer to the general knock-in approach (e.g. *Htt* CAG knock-in) and to the precise size of the expanded CAG repeat allele, which varies in any given progeny due to intergenerational CAG repeat instability. Thus, the actual genotyped *Htt* CAG repeat size of the parental Hdh^Q111^ mice was 139 CAG repeats. As shown in Fig. 1, crosses between Hdh^Q111^ knock-in and wild type (CSS10 and B6J) mice were made in one direction—Hdh^Q111/+^ male × B6J and CSS10 females—in order to control for possible parental effects of the *Htt* mutant allele. Briefly, male heterozygous Hdh^Q111/+^ knock-in mice were crossed with CSS10 female mice in order to generate wild type Hdh^+/+^.C57BL/6J.Chr10^A/J^ (Hdh^+/+^B6J.AJ10) and mutant Hdh^Q111/+^.C57BL/6J.Chr10^A/J^ (Hdh^Q111/+^B6J.AJ10) mice that carried one chr10 from strain AJ on an otherwise B6J background. Subsequently, the same male heterozygous Hdh^Q111/+^ knock-in mice were crossed with B6J female mice in order to generate wild type Hdh^+/+^.C57BL/6J (Hdh^+/+^B6J) and mutant Hdh^Q111/+^.C57BL/6J (Hdh^Q111/+^B6J) mice on a complete B6J background. Animal experiments were performed to minimise pain and discomfort, under an approved protocol of the Massachusetts General Hospital Subcommittee on Research Animal Care.

### Experimental design

From the F1 offspring mice (*n* = 119), we selected two different sets of mice for phenotypic somatic instability (set 1) and immunohistochemistry assays (set 2), with groups consisting of both female and male, *Htt* mutant and wild type mice from both B6J.AJ10 and B6J crosses (Supplementary Table 1). Set 1 (*n* = 20) consisted of ten 5-month-old *Htt* mutant mice per genotype (Hdh^Q111/+^B6J.AJ10 and Hdh^Q111/+^B6J) matched by gender (5 female and 5 male per genotype) and *Htt* CAG repeat size. Set 2 (*n* = 40) consisted of ten 5-month-old mice per genotype (Hdh^Q111/+^B6J.AJ10, Hdh^Q111/+^B6J, Hdh^+/+^B6J.AJ10 and Hdh^+/+^B6J) matched by gender (5 females and 5 males per genotype) and *Htt* CAG repeat size. All *Htt* mutant mice used in this study were heterozygous for the *Htt* CAG mutation. As we could not generate data for all the animals in all assays, the number of mice included into the analyses of each test is given in the legends.

### Body weight

F1 offspring mice were fed Prolab Isopro RMH 3000 (PMI Nutrition International, Brentwood, MO) ad libitum and weighed at weekly intervals from the time of weaning (3 weeks) until 5 months (~24 weeks). The following body weight traits were analysed: initial (IW: weight in grams at ~25 days of age), medium (MW: weight in grams at ~98 days of age) and final weight (FW: weight at ~169 days of age). We also calculated the mean weight gain/day in grams for the first (EWG) and last (FWG) ~72 days and the weight gain per day in grams/day (WG), as previously described (Burrage et al. 2010).

### Genotyping and analysis of somatic instability

Genomic DNA was isolated from fresh-frozen collected tissues (tail, striatum and liver) using DNeasy Blood & Tissue kit (Qiagen, Valencia, CA). Genotyping of the Hdh^Q111^ knock-in allele was carried out using a previously established polymerase chain reaction (PCR) amplification assay, with fluorescently labelled primers (Wheeler et al. 2003). The size of the PCR products was then determined using the ABI PRISM 3730*xl* automated DNA Sequencer (Applied Biosystems, Foster City, CA) and GeneMapper version 3.7 software. All runs included the same control DNAs of known *Htt* CAG repeat size. Somatic instability was quantified from GeneMapper traces as described previously (Lee et al. 2010). Briefly, the main allele was identified as the highest peak in each analysis and peaks with height less than 20 % of the main allele were excluded. The peak height of each peak was divided by the sum of the heights of all signal peaks and then multiplied by the CAG change relative to the main allele. These values were summed to generate an instability index. To qualitatively assess the different patterns of repeat instability in liver and striatum, the distance (in CAG repeats) between the two modes and the distance (in CAG repeats) between the constitutive repeat mode and the longest repeat was calculated as previously described (Lee et al. 2011).

### Immunohistochemistry

Coronal sections of paraformaldehyde-perfused and post-fixed, gelatin-embedded hemisphere brains were performed at Neurosciences Associates (NSALabs, Knoxville, TN) using MultiBrain Technology. With this technology, all mouse brain hemispheres were embedded in one single block that was freeze-sectioned at 35 µm in the coronal plane (throughout the striatum) with a sliding microtome, resulting in free-floating sections. Designated sections across the striatum were stained with either EM48 and thionine counterstain or DARPP-32 at 210-µm intervals. Bright field microscopy was performed with an Olympus BX51 microscope equipped with a Qcolor5 Olympus camera and Qcapture image acquisition software. Images that were to be quantified and compared were taken with the same exposure times. For the EM48 sections, three micrographs (×40 objective) were taken from the dorsal medial striatum in three consecutive sections from each mouse. The number of nuclear huntingtin inclusions was quantified in three striatal areas per mouse and normalised to the number of EM48-positive cells. For the DARPP-32 sections, micrographs (×4 objective) in three consecutive sections were taken from each mouse and DARPP-32 intensity was quantified using ImageJ image analysis software (Schneider et al. 2012).

### Statistical analyses

Pairwise comparisons were determined using an unpaired *t* test or a nonparametric unpaired Mann–Whitney test (for data that did not display a normal distribution). Statistical analyses were performed using GraphPad Prism 6.00 (GraphPad Software, La Jolla, CA). Sample size estimation (power = 0.80, *α* = 0.05) was calculated using the R package samplesize (http://CRAN.R-project.org/package=samplesize).

## Results

### Genetic variance between B6J and AJ strains at mouse chr10

**Fig. 2:**
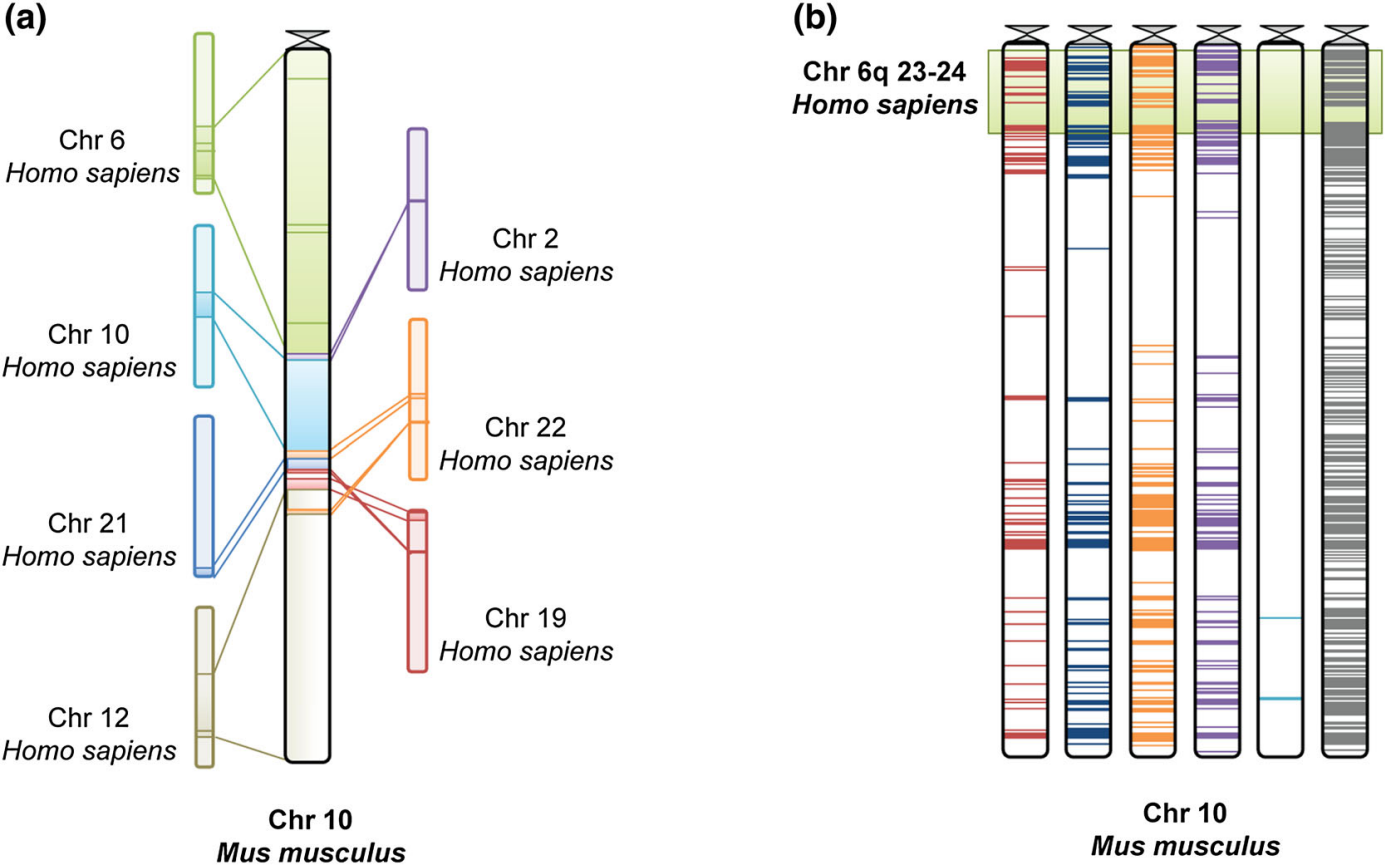
Genetic architecture of the ~131 Mbp mouse chr10. **a** Representative homology scheme of mouse chr10 compared with human. **b** Location of the reported genetic variants between B6J and AJ strains at mouse chr10. Coding non-synonymous variants are represented in *red*, coding synonymous in *dark blue*, *locus*-region in *orange*, mRNA-UTR in *purple*, splice-site in *light blue* and intronic in *grey* (Color figure online)

A genome wide HD modifier linkage scan has provided strong evidence for a QTL, between markers D6S1009 and D6S2436, at the human 6q23–24 interval, that modulates age at HD neurological onset (Li et al. 2003, 2006). This region (~16 Mbp) includes at least 80 different human genes that corresponded to 72 homologue genes in the mouse, as annotated in the Ensembl Genome Browser (Flicek et al. 2013). The human 6q23–24 interval is in synteny with part of mouse chr10 (Fig. 2a), comprising 71 of the homologue genes. According to the Mouse Genome Informatics database (Eppig et al. 2012), B6J and AJ mice have about 16,000 allelic differences at chr10 (~131 Mbp): 106 non-synonymous, 205 synonymous, ~14,600 intronic, 576 locus-region (in an untranscribed region flanking a gene: 2 Kb 5′ or 0.5 Kb 3′), 316 mRNA-UTR (in the transcript but not in the coding region interval) and 3 splice-site (in the first or two last bases of an intron) variants (Fig. 2b). From these variants, 19 coding non-synonymous SNPs, most likely to have functional impact, are located in 12 homologues of human genes located at 6q23–24: *Mthfd1* *l, 1700052N19Rik*, *Katna1*, *Sash1*, *Samd5*, *Epm2a*, *Utrn*, *Stx11*, *Hivep2*, *Nhsl1*, *Tnfaip3* and *Ifngr1*.

To determine whether allelic differences at any of these genes or other loci across chr10 may strongly alter dominant consequences of the expanded CAG repeat at the *Htt* locus in heterozygous Hdh^Q111^ mice, we bred the latter with either CSS10 mice or with B6J mice as described in “Materials and methods.” For this range-finding study, the cohorts of F1 progeny were phenotyped at about 5 months of age by monitoring the rate of body weight gain, previously reported to be associated with B6J.AJ chr10 variants, and five outcomes of the Hdh^Q111^ allele: somatic CAG repeat instability in the striatum and in the liver (Lee et al. 2010, 2011; Wheeler et al. 1999), the appearance of diffuse nuclear huntingtin stain and intranuclear huntingtin inclusions in medium size spiny striatal neurons (Wheeler et al. 2002) and a novel phenotype, the decrease in DARPP-32 levels in the striatum. At 5 months of age, the majority of these CAG-dependent phenotypes were expected from previous experience to be fully penetrant on the B6J genetic background, minimising the number of mice of each genotype to be evaluated. F1 cohorts of ~ten mice of each genotype (five males and five females) was chosen as a reasonable starting point for assessing the possibility of dominant variants of strong effect that would become apparent with a subset of each cohort in each assay.

### Body weight

**Fig. 3:**
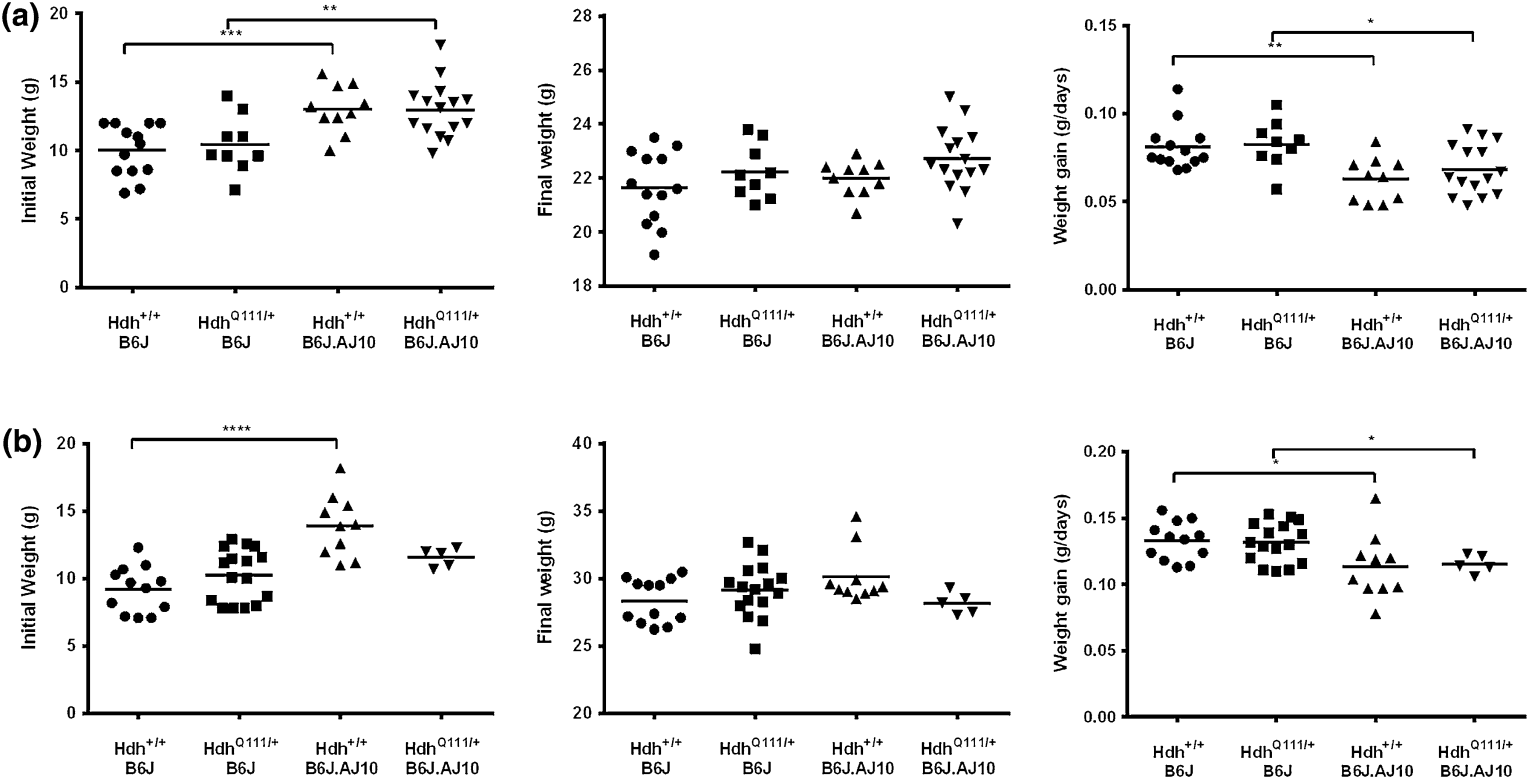
One single copy of AJ chr10 has an effect on the patterns of weight gain. Scatter plots represent the individual values of the initial weight (at ~3 weeks of age), the final weight (at ~24 weeks of age) and the mean weight gain per day for **a** female and **b** male mice. The mice used for the body weight analysis are as follows: Hdh^+/+^B6J (13 females and 14 males), Hdh^Q111/+^B6J (9 females and 16 males), Hdh^+/+^B6J.AJ10 (10 females and 10 males) and Hdh^Q111/+^B6J.AJ10 (15 females and 5 males). Line represents mean of values for each genotype. ****p < 0.0001, ***p < 0.001, **p < 0.01 and *p < 0.05

We examined the rate of body weight gain as a primary endpoint because it has been reported, from phenotyping across the members of the entire CSS panel, that dominant AJ chr10 variants dampen the rapid rate of body weight gain of B6J mice, in F1 B6J.AJ10 mice (Burrage et al. 2010). Furthermore, in HD subjects weight loss is often observed in the course of the disease (Aziz et al. 2008; Djousse et al. 2002; Farrer and Yu 1985; Robbins et al. 2006; Sanberg et al. 1981) despite normal to very high caloric intake (Marder et al. 2009; Morales et al. 1989; Trejo et al. 2004). It is correlated with disease progression (Myers et al. 1991) and patients with higher expanded CAG repeat exhibit more rapid loss of body weight (Aziz et al. 2008). In the knock-in Hdh^Q111^ mice (on a CD1 background), homozygous males had a lower body weight than wild type and heterozygous mice at 28 weeks (Menalled et al. 2009). Therefore, to assess the potential effect of the Hdh^Q111^ mutation on a B6J background and of the different test genetic backgrounds (chr10^B6J/B6J^ vs chr10^B6J/AJ^), we analyzed body weight at three different ages (3, 14, and 24 weeks). As shown in Fig. 3, wild type B6J mice showed significant lower body weight than wild type B6J.AJ10 mice at 3 weeks of age for both genders (*p* = 0.0008, mean IW ± SD: 10.02 ± 1.89 vs 13.03 ± 1.74 for females and *p* < 0.0001, mean IW ± SD: 9.22 ± 1.72 vs 13.92 ± 2.29 for males). However, they tended to gain more weight per day than B6J.AJ10 mice (*p* = 0.0026, mean WG ± SD: 0.081 ± 0.013 vs 0.063 ± 0.012 for females and *p* = 0.0302, WG ± SD: 0.133 ± 0.014 vs 0.113 ± 0.024 for males), resulting in similar body weights between B6J and B6J.AJ10 mice by week 24 (mean FW ± SD: 21.64 ± 1.35 vs 21.99 ± 0.64 for females, and 28.35 ± 1.63 vs 30.13 ± 2.03 in males, respectively). Therefore, the presence of the AJ10 chromosome was associated with less rapid weight gain, thereby confirming an earlier phenotypic effect on an otherwise B6J background. However, there was no significant difference in weight at any time point between Hdh^Q111^ mutant and wild type mice, for either of the two different chr10 genetic backgrounds.

### CAG instability of Hdh^Q111^ knock-in alleles

**Fig. 4:**
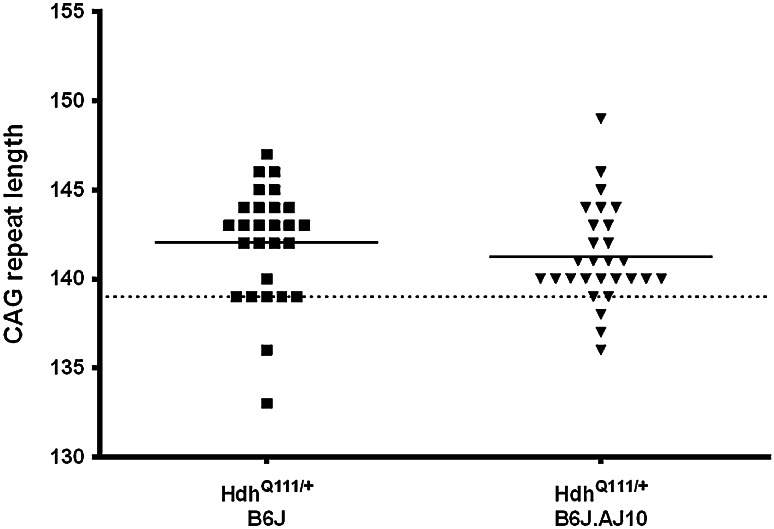
CAG repeat size of the knock-in alleles in Hdh^Q111/+^B6J and Hdh^Q111/+^B6J.AJ10 progeny. The mean CAG repeat size of the mice used in this study was 142.0 ± 3.2 for Hdh^Q111/+^B6J (*n* = 27) and 141.3 ± 2.8 for Hdh^Q111/+^B6J.AJ10 (*n* = 28). *Solid line* represents mean of CAG repeat for each genotype while the *dashed line* represents the CAG repeat size transmitted from the parental Hdh^Q111/+^B6J mice (139 CAGs)

Previous studies have shown that in Hdh^Q111^ knock-in mice intergenerational CAG repeat length changes with a paternal expansion bias (Lloret et al. 2006; Wheeler et al. 1999). In our study, the *Htt* mutant allele was transmitted from male Hdh^Q111/+^ knock-in mice with the same background (B6J) and constitutional (i.e. in tail DNA) CAG repeat length (139 CAG repeats). Therefore, as expected, we observed a paternal expansion bias (74 % in crosses of Hdh^Q111/+^ mice with B6J and 82 % in crosses with CSS10 mice), but no significant repeat length difference in the transmissions of the *Htt* 139 CAG allele to Hdh^Q111/+^B6J (mean ± SD = 3.04 ± 3.18) and Hdh^Q111/+^B6J.AJ10 mice (mean ± SD = 2.25 ± 2.77) was detected. Figure 4 shows the actual CAG repeat size of the Hdh^Q111^ knock-in allele in both Hdh^Q111/+^B6J and Hdh^Q111/+^B6J.AJ10 progeny used in this study.

**Fig. 5:**
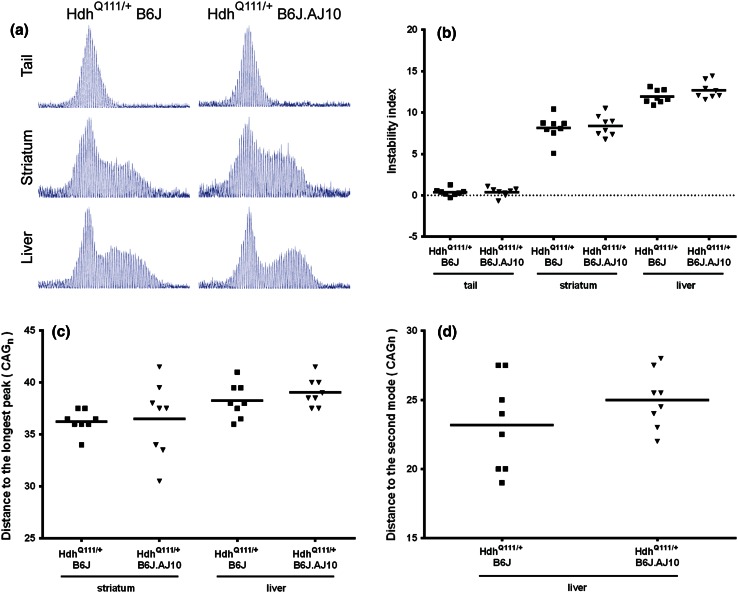
Hdh^Q111/+^B6J.AJ10 mice showed a mild increase of liver somatic repeat instability when compared to Hdh^Q111/+^B6J mice. **a** GeneMapper traces of PCR-amplified *Htt* CAG repeats from tail, striatum and liver of representative 5 months Hdh^Q111/+^B6J (144 CAGs) and Hdh^Q111/+^B6J.AJ10 (141 CAGs) mice. **b** Somatic repeat instability was quantified from GeneMapper traces by determining an instability index for tail, striatum and liver of each mouse. Additionally, to capture the different patterns of repeat instability in liver and striatum, we measured **c** the distance between the constitutive repeat mode and the longest repeat after background correction and **d** the distance between the modes of the constitutive and somatically expanded repeats. Hdh^Q111/+^B6J (*n* = 8) mice are represented in *squares* and Hdh^Q111/+^B6J.AJ10 (*n* = 8) mice in *triangles*

Hdh^Q111/+^ knock-in mice also exhibit CAG length, age dependent and tissue specific somatic instability, with significant accumulation of expansions in striatum and liver that becomes apparent by 5 months of age (Lee et al. 2010, 2011; Wheeler et al. 1999). In order to evaluate the potential effect of AJ-B6J genetic variants at chr10 in somatic instability, we extracted genomic DNA from one stable (tail) and two unstable (liver and striatum) tissues from Hdh^Q111/+^B6J and Hdh^Q111/+^B6J.AJ10 mice at 5 months of age. As shown in Fig. 5a, the *Htt* CAG repeats in tail were very stable in contrast to striatum and liver that showed significant levels of instability, irrespective of chr10 background. Next we quantified the levels of repeat instability for each tissue using a conservative threshold (20 % of the highest peak). As shown in Fig. 5b, the highest instability indices were observed in the liver (+11.95 ± 0.81 for Hdh^Q111/+^B6J and +12.71 ± 1.03 for Hdh^Q111/+^B6J.AJ10) followed by the striatum (+8.15 ± 1.51 for Hdh^Q111/+^B6J and +8.40 ± 1.27 for Hdh^Q111/+^B6J.AJ10). There was no significant difference for the instability indices, or for the expansion and contraction indices (data not shown) when comparing the two groups of Hdh^Q111^ mice. As previously described (Lee et al. 2011), the patterns of repeat instability differed between liver and striatum: while in the liver a distinct population of unstable CAG repeats was evident, in the striatum these repeats were more broadly distributed. To qualitatively characterise these different patterns of repeat instability, two additional measurements were made: the distance to the longest repeat (striatum and liver, Fig. 5c) and the distance between the two modes (liver, Fig. 5d). There was no significant difference in the patterns of repeat instability when comparing Hdh^Q111/+^B6J and Hdh^Q111/+^B6J.AJ10. However, as for the instability index, we did observe a numerically larger distribution of the unstable repeats in the liver of Hdh^Q111/+^B6J.AJ10 mice.

### Nuclear mutant huntingtin

**Fig. 6:**
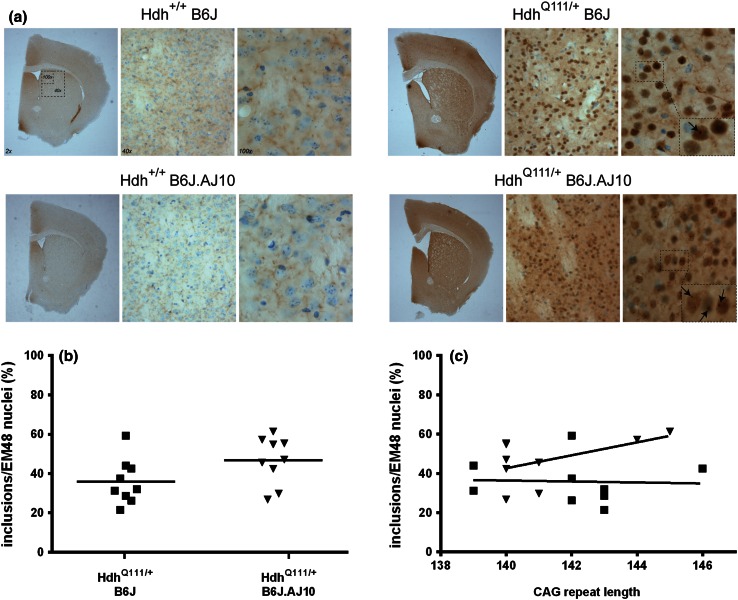
Hdh^Q111/+^B6J.AJ10 mice showed a mild increase of neuronal intranuclear inclusions when compared to Hdh^Q111/+^B6J mice. **a** Micrographs of striata stained with EM48 and counterstained with thionine of representative 5 months mice with Hdh^+/+^B6J, Hdh^Q111/+^B6J (139 CAGs), Hdh^+/+^B6J.AJ10 and Hdh^Q111/+^B6J.AJ10 (140 CAGs) genotypes. **b** Quantification of the percentage of EM48-positive cells containing an inclusion (*line* represents mean of values for each genotype) and **c** its correlation with constitutive *Htt* CAG repeat size (the best fit linear regression line of inclusion/EM48 nuclei by CAG repeat size for Hdh^Q111/+^B6J is represent with *solid line* and for Hdh^Q111/+^B6J.AJ10 with the *thinner solid line*). Hdh^Q111/+^B6J (*n* = 9) mice are represented in *squares* and Hdh^Q111/+^B6J.AJ10 (*n* = 9) mice in *triangles*. *Each individual value* represents the mean observed on three consecutive striatal sections for each mouse

 Another early, dominant, CAG-length-dependent phenotype in Hdh^Q111^ mice is the time-dependent immunostaining of mutant huntingtin in the nuclei of striatal neurons. Previous studies have detected early (~2.5 months) diffuse-immunostaining nuclear mutant huntingtin and later (6–12 months) intranuclear inclusions of mutant huntingtin amino-terminal fragments, using the anti-huntingtin antibody EM48 (Lloret et al. 2006; Wheeler et al. 2000, 2002). In order to determine the effect of the chr10 background on the nuclear mutant huntingtin phenotype, we immunostained striatal sections from 5-month mice with EM48 antibody (Fig. 6a). As expected, EM48 staining was not detected in the wild type mice (Fig. 6a, right panel). When comparing *Htt* CAG knock-in mice, we found a similar number of nuclei with diffuse mutant huntingtin stain (data not shown) and some nuclei exhibited intranuclear inclusions (Fig. 6a, left panel). We found that, compared to Hdh^Q111/+^B6J mice, the percentage of EM48-positive nuclei with intranuclear inclusions was higher in Hdh^Q111/+^B6J.AJ10 mice (35.88 ± 11.45 vs 46.69 ± 12.11, respectively), although the difference did not reach statistical significance (Fig. 6b; *p* = 0.0694). Moreover, the number of intranuclear inclusions was found to depend on the constitutive CAG repeat length of Hdh^Q111/+^B6J.AJ10 mice, with longer CAG lengths resulting in an increased percentage of EM48 nuclei with inclusions (and higher when compared with Hdh^Q111/+^B6J mice with similar CAG repeat length) (Fig. 6c).

### DARPP-32 levels in striatial medium spiny neurons

**Fig. 7:**
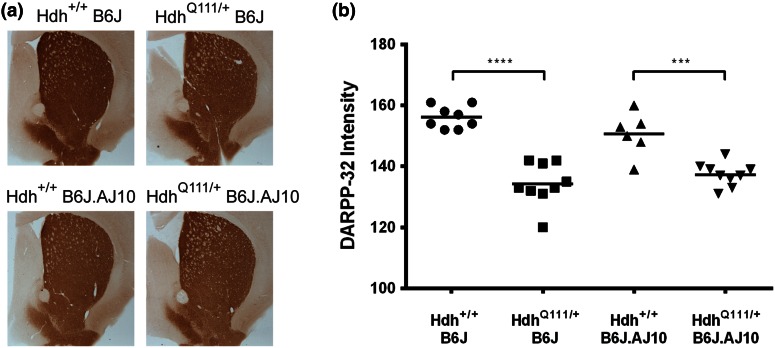
Hdh^Q111/+^ knock-in mice showed a dramatic decrease of DARPP-32 immunoreactivity when compared to wild type mice. **a** Micrographs of DARPP-32 positive cells in the striata and nucleus accumbens of representative 5 months mice with Hdh^+/+^B6J, Hdh^Q111/+^B6J (142 CAGs), Hdh^+/+^B6J.AJ10 and Hdh^Q111/+^B6J.AJ10 (140 CAGs) genotypes. **b** Quantification of DARPP-32 intensity (arbitrary values ranging from 0 to 255). The mice used for the DARPP32 analysis are as follows: Hdh^+/+^B6J (*n* = 8), Hdh^Q111/+^B6J (*n* = 9), Hdh^+/+^B6J.AJ10 (*n* = 6) and Hdh^Q111/+^B6J.AJ10 (*n* = 9). *Each individual value* represents the mean observed on three consecutive striatal sections for each mouse. *Line* represents mean of values for each genotype. ****p < 0.0001 and ***p < 0.001

The striatum is critically affected in HD patients, where there is a selective and progressive loss of MSNs (Vonsattel and DiFiglia 1998; Vonsattel et al. 1985). DARPP-32, a dopamine D1 receptor-activated molecule that regulates phosphatase and kinase activity, is a marker of these striatal neurons. Levels of DARPP-32 were substantially reduced in the striatal MSNs of multiple HD mouse models (Bibb et al. 2000; Hickey et al. 2008; Slow et al. 2003; van Dellen et al. 2000). To assess whether the *Htt* CAG mutation may produce this abnormal DARPP-32 phenotype on a B6J background, we immunostained striatal sections of 5 months of age mice with DARPP-32 antibody (Fig. 7a). DARPP-32 immunoreactivity was significantly reduced (*p* < 0.0001) in the striatum (and nucleus accumbens) of Hdh^Q111/+^B6J (134.2 ± 7.1) by 14 % when compared with Hdh^+/+^B6J control littermates (156.0 ± 3.7) (Fig. 7b). A similar, though milder, decrease was observed in mice carrying one copy of the AJ chr10, with Hdh^Q111/+^B6J.AJ10 (137.4 ± 3.7) mice showing a 9 % reduction (*p* = 0.0003) when compared with control littermates (150.7 ± 7.0). However, we did not find any significant effect of the chr10 background in the levels of DARPP-32 when comparing B6J with B6J.10 mice, for both wild type (*p* = 0.0920) and mutant (*p* = 0.2521) *Htt* genotypes. It should be noted, however, that Hdh^+/+^B6J.AJ10 showed a 3.5 % decrease of DARPP-32 levels when compared with wild type B6J mice so that the similar DARPP-32 immunoreactivity signal in Hdh^Q111/+^B6J.AJ10 and Hdh^Q111/+^B6J striatal sections may reflect a slower rate of decrease in DARPP-32 staining in the former than the latter genotype.

## Discussion

The length of the *HTT* CAG repeat is the critical determinant of HD pathogenesis. However, the wide variability in the particular symptoms that present at diagnosis, and their timing, provide evidence that the CAG-initiated disease process can be modified. The historical candidate gene approach to identifying modifier loci, where genes in pathways thought to be involved in HD pathogenesis are individually tested for an effect on AO, has produced inconsistent findings and is being overtaken by unbiased methods that can test millions of human genetic variants across the entire genome in cohorts of HD subjects. Linkage studies have identified possible chromosomal regions containing modifier genes, including a confirmed large interval on chromosome 6q (Gayan et al. 2008; Li et al. 2003, 2006) and genome wide association studies to detect effects of modifiers of motor onset in HD subjects are ongoing. The success of genome wide approaches make the development of strategies that can efficiently triage variants and genes located within an implicated region to find the one (or two) that are responsible for the modifying effect a high priority. A functional in vivo approach that could disclose a modifier of the CAG-disease process, including gene variants whose effects may only be evident in presence of the HD mutation, would provide a powerful complement to other discovery methods in studies with HD subjects, from natural history studies and with the advent of induced pluripotent stems cell technologies, in cell culture (The HD iPSC Consortium 2012).

We set a high bar in exploring a novel approach with CSS and Hdh^Q111/+^ CAG repeat knock-in mice in a range-finding study that utilised variants on murine chr10 synteny to evaluate the human 6q23–24 HD modifier linkage region. In modest cohorts of mice, we tested, simultaneously, whether any of the thousands of B6J versus AJ chr10 variants, including 19 coding non-synonymous SNPs, most likely to have functional impact, located in 12 homologues of human genes in the linkage region (*Mthfd1l, 1700052N19Rik*, *Katna1*, *Sash1*, *Samd5*, *Epm2a*, *Utrn*, *Stx11*, *Hivep2*, *Nhsl1*, *Tnfaip3*, *Ifngr1*), may act as dominant modifiers (of strong effect) of early outcomes of the dominant CAG expansion-dependent disease process in heterozygous of Hdh^Q111/+^ mice. We selected disease phenotypes shown to be modifiable by genetic background (B6 vs 129) (Lloret et al. 2006) and minimised extraneous factors by generating two sets of F1 Hdh^Q111^ knock-in mice with identical CAG repeat lengths (~141/142 ± 3 CAGs) and genetic background (B6J), except for the chr10 in the B6J.AJ10 mice that carried one chromosome from the AJ strain.

Dominant functional chr10^A/J^ variants, whose effects did not require the presence of the *Htt* CAG repeat allele to be observed, were segregating in our F1 progeny. We replicated a previously reported metabolic effect of chr10^A/J^ variants in moderating the rapid B6J weight gain associated with an A/J obesity-resistance QTL at chr10 (Burrage et al. 2010; Singer et al. 2004), though those mice were on highfat diet and ours were on regular chow. However, in the cohorts of Hdh^Q111/+^ mice analyzed we did not observe dominant chr10^A/J^ modifiers of CAG-dependent phenotypes that were as strong as the variant/s responsible for dampening the rate of weight gain. Our results, therefore, do not provide overwhelming independent validation of the modifier region implicated by the 6q23–24 linkage peak.

There are several possible reasons that clearly statistically significant modifying effects on Hdh^Q111^ CAG repeat phenotypes may not have emerged from our study. The first is cohort size. Our study design attempted to minimise extraneous factors by generating all F1 animals for the study at once from the same parents but post-mortem the F1 progeny were assigned to one of two groups for efficient phenotyping in each of the two general assay formats (CAG instability and immunohistochemistry). Therefore, the cohorts for the CAG-dependent phenotypes (*n* = 10) were half the size of the cohorts assessed for body weight. Based on our current data, we would need at least 19 mice per group to detect the small effect of the chr10^A/J^ variants in the traits approaching significance at 5 months of age (somatic instability in the liver and intranuclear inclusions), perhaps fewer at a more advanced age where each phenotype is more robustly detected (Wheeler et al. 2002). Second, the gene(s) with the modifying effects revealed by linkage in HD subjects may not be captured by the natural chr10 variation that distinguishes the AJ and B6J strains, or if the true modifier has been tested in our study then its effects in HD subjects may be unrelated to early outcomes of the CAG expansion that we monitored in Hdh^Q111^ mice. On the other hand, our findings provide suggestive (though not statistically significant) evidence for modifying effects of chr10 variants on some of the Hdh^Q111^ CAG disease phenotypes, in particular those involved in liver somatic CAG repeat instability and striatal neuron nuclear-aggregate formation. Indeed, we found that the different genetic background does not seem to have an effect on striatal instability but the AJ chr10 might have a mild detrimental effect on the levels and pattern of repeat instability in the liver, as the same trend was observed in all traits analyzed for this tissue (instability index, distance to the longest peak and to the second mode). Moreover, even though the effect of the AJ chr10 on striatum CAG instability was not statistically significant, the Hdh^Q111/+^B6J.AJ10 mice seemed to have a slightly higher number of neuronal inclusions than Hdh^Q111/+^B6J, despite similar levels of neuronal change in the striatum (DARPP-32 levels). An improved high-resolution quantitative method for detecting EM48-aggregation foci may be required to better assess the impact of modifiers of this phenotype. It is noteworthy that the reduction of DARPP-32 levels from wild type to *Htt* mutant mice was not as accentuated in Hdh^Q111/+^B6J.AJ10 as the observed for Hdh^Q111/+^B6J, suggesting that the effect of the *Htt* CAG mutation in decreasing the DARPP-32 stain intensity is greater than the effect of the difference in genetic background (of a single copy of chr10) on this phenotype. Indeed, supporting this interpretation, *Htt* CAG alleles of about 140 repeats (comparable to the mean CAG size of our DARPP32 cohort) dramatically decrease *Ppp1r1b* (alias Darpp32; ENSMUSG00000061718) mRNA levels in 6-month heterozygote B6J *Htt* knock-in mouse striatum, compared to wild-type littermates (*t* test, *p* = 0.000059), as revealed by inspection of summary whole genome RNA sequencing data from a B6J *Htt* CAG repeat knock-in mouse allelic series available from the CHDI Foundation website (http://chdifoundation.org/datasets/).

These trends, therefore, suggest that chr10 variants may modify some CAG-associated phenotypes but with effect sizes too small to be reliably detected at the ages and cohort sizes that we utilised. If the trends that we have observed do reflect true modification, with cohorts of sufficient size and age, validation and fine-structure mapping of potential QTL(s) at chr10 could be achieved by using sub-strain chr10 consomic mice with the region syntenous to 6q23–24. In general, therefore, this unbiased cross-species CCS mouse strain approach may offer an efficient in vivo route to prioritise (conserved) genes within chromosome regions that contain dominant modifier loci of relatively strong effect that conform to the genetic criteria of the *HTT* CAG repeat mechanism in HD subjects (dominant, CAG length dependent and with striatal specificity).

## Electronic supplementary material

Below is the link to the electronic supplementary material.

Supplementary material 1 (DOCX 98 kb)
