# Supplementary material for: Chromosome substitution strain assessment of a Huntington’s disease modifier locus
Source: Mamm Genome. 2015 Feb 3;26(3):119–30. doi: 10.1007/s00335-014-9552-9 (PMC4372682; doi:10.1007/s00335-014-9552-9)
Supplement: Supplementary file 1 — Supplementary material 1 (DOCX 98 kb) [file 335_2014_9552_MOESM1_ESM.docx]

##### **SUPPLEMENTARY DATA**

**Supplementary Table 1.** List of mice used for each phenotype assay and their characteristics (group, gender and *Htt* CAG repeat).

| **Mouse** | **Chr10** | **Gender** | ***Htt* CAG**  **repeat** | **Body**  **Weight** | **Somatic**  **Instability** | **EM48** | **DARPP-32** |
| --- | --- | --- | --- | --- | --- | --- | --- |
| Q1558 | B6J/B6J | F | WT | Yes |  | N.A. | Yes |
| Q1853 | B6J/B6J | F | WT | Yes |  | N.A. | Yes |
| R1441 | B6J/B6J | F | WT | Yes |  | N.A. | Yes |
| R1124 | B6J/B6J | F | WT | N.A. |  | N.A. | Yes |
| R1444 | B6J/B6J | F | WT | Yes |  | x | x |
| R1442 | B6J/B6J | F | WT | N.A. |  | x | x |
| 926 | B6J/B6J | F | WT | Yes |  |  |  |
| 929 | B6J/B6J | F | WT | Yes |  |  |  |
| 931 | B6J/B6J | F | WT | Yes |  |  |  |
| 932 | B6J/B6J | F | WT | Yes |  |  |  |
| Q1555 | B6J/B6J | F | WT | Yes |  |  |  |
| Q1557 | B6J/B6J | F | WT | Yes |  |  |  |
| Q1559 | B6J/B6J | F | WT | Yes |  |  |  |
| Q1855 | B6J/B6J | F | WT | Yes |  |  |  |
| R1125 | B6J/B6J | F | WT | N.A. |  |  |  |
| R1126 | B6J/B6J | F | WT | N.A. |  |  |  |
| R1127 | B6J/B6J | F | WT | N.A. |  |  |  |
| Q1562 | B6J/B6J | F | WT | Yes |  |  |  |
| Q1566 | B6J/B6J | M | WT | Yes |  | N.A. | Yes |
| Q1947 | B6J/B6J | M | WT | Yes |  | N.A. | Yes |
| R1449 | B6J/B6J | M | WT | Yes |  | N.A. | Yes |
| R1453 | B6J/B6J | M | WT | Yes |  | N.A. | Yes |
| R1446 | B6J/B6J | M | WT | Yes |  | x | x |
| Q1563 | B6J/B6J | M | WT | Yes |  |  |  |
| Q1862 | B6J/B6J | M | WT | Yes |  |  |  |
| Q1866 | B6J/B6J | M | WT | Yes |  |  |  |
| Q1948 | B6J/B6J | M | WT | Yes |  |  |  |
| Q1953 | B6J/B6J | M | WT | Yes |  |  |  |
| Q1956 | B6J/B6J | M | WT | Yes |  |  |  |
| R1196 | B6J/B6J | M | WT | Yes |  |  |  |
| R1197 | B6J/B6J | M | WT | Yes |  |  |  |
| R1455 | B6J/B6J | M | WT | Yes |  |  |  |
| 928 | B6J/B6J | F | 139 | Yes | Yes |  |  |
| 934 | B6J/B6J | F | 139 | Yes | Yes |  |  |
| 930 | B6J/B6J | F | 142 | Yes | Yes |  |  |
| 933 | B6J/B6J | F | 143 | Yes | Yes |  |  |
| Q1854 | B6J/B6J | F | 145 | Yes | Yes |  |  |
| Q1556 | B6J/B6J | F | 139 | Yes |  | Yes | Yes |
| Q1856 | B6J/B6J | F | 142 | Yes |  | Yes | Yes |
| Q1945 | B6J/B6J | F | 143 | Yes |  | Yes | Yes |
| R1123 | B6J/B6J | F | 139 | N.A. |  | Yes | Yes |
| Q1561 | B6J/B6J | F | 144 | Yes |  |  |  |
| Q1859 | B6J/B6J | M | 133 | Yes | Yes |  |  |
| R1451 | B6J/B6J | M | 136 | Yes | Yes |  |  |
| Q1861 | B6J/B6J | M | 144 | Yes | Yes |  |  |
| Q1949 | B6J/B6J | M | 144 | Yes | Yes |  |  |
| Q1988 | B6J/B6J | M | 145 | Yes | Yes |  |  |
| Q1864 | B6J/B6J | M | 142 | Yes |  | Yes | Yes |
| Q1867 | B6J/B6J | M | 142 | Yes |  | Yes | Yes |
| Q1565 | B6J/B6J | M | 143 | Yes |  | Yes | Yes |
| Q1858 | B6J/B6J | M | 143 | Yes |  | Yes | Yes |
| Q1865 | B6J/B6J | M | 146 | N.A. |  | Yes | Yes |
| R1454 | B6J/B6J | M | 139 | Yes |  |  |  |
| R1450 | B6J/B6J | M | 140 | Yes |  |  |  |
| R1447 | B6J/B6J | M | 143 | Yes |  |  |  |
| R1448 | B6J/B6J | M | 143 | Yes |  |  |  |
| Q1950 | B6J/B6J | M | 144 | Yes |  |  |  |
| R1452 | B6J/B6J | M | 146 | Yes |  |  |  |
| Q1989 | B6J/B6J | M | 147 | Yes |  |  |  |
| Q1683 | B6J/AJ | F | WT | Yes |  | N.A. | Yes |
| Q1757 | B6J/AJ | F | WT | Yes |  | N.A. | Yes |
| Q1758 | B6J/AJ | F | WT | Yes |  | N.A. | Yes |
| R1467 | B6J/AJ | F | WT | Yes |  | x | x |
| R1494 | B6J/AJ | F | WT | Yes |  | x | x |
| Q1679 | B6J/AJ | F | WT | Yes |  |  |  |
| Q1756 | B6J/AJ | F | WT | Yes |  |  |  |
| Q1759 | B6J/AJ | F | WT | Yes |  |  |  |
| Q1958 | B6J/AJ | F | WT | Yes |  |  |  |
| R1497 | B6J/AJ | F | WT | Yes |  |  |  |
| Q1794 | B6J/AJ | F | WT | N.A. |  |  |  |
| Q1795 | B6J/AJ | F | WT | N.A. |  |  |  |
| Q1796 | B6J/AJ | F | WT | N.A. |  |  |  |
| Q1797 | B6J/AJ | F | WT | N.A. |  |  |  |
| R1602 | B6J/AJ | F | WT | N.A. |  |  |  |
| R1603 | B6J/AJ | F | WT | N.A. |  |  |  |
| Q1684 | B6J/AJ | M | WT | Yes |  | N.A. | Yes |
| R1605 | B6J/AJ | M | WT | N.A. |  | N.A. | Yes |
| R1920 | B6J/AJ | M | WT | N.A. |  | N.A. | Yes |
| R1473 | B6J/AJ | M | WT | Yes |  | x | x |
| R1498 | B6J/AJ | M | WT | Yes |  | x | x |
| Q1686 | B6J/AJ | M | WT | Yes |  |  |  |
| Q1760 | B6J/AJ | M | WT | Yes |  |  |  |
| Q1761 | B6J/AJ | M | WT | Yes |  |  |  |
| Q1762 | B6J/AJ | M | WT | Yes |  |  |  |
| R1868 | B6J/AJ | M | WT | Yes |  |  |  |
| R1869 | B6J/AJ | M | WT | Yes |  |  |  |
| R1870 | B6J/AJ | M | WT | Yes |  |  |  |
| Q1798 | B6J/AJ | M | WT | N.A. |  |  |  |
| Q1799 | B6J/AJ | M | WT | N.A. |  |  |  |
| R1607 | B6J/AJ | M | WT | N.A. |  |  |  |
| R1917 | B6J/AJ | M | WT | N.A. |  |  |  |
| R1495 | B6J/AJ | F | 138 | Yes | Yes |  |  |
| R1493 | B6J/AJ | F | 139 | Yes | Yes |  |  |
| Q1959 | B6J/AJ | F | 140 | Yes | Yes |  |  |
| Q1753 | B6J/AJ | F | 144 | Yes | Yes |  |  |
| R1601 | B6J/AJ | F | 141 | N.A. | Yes |  |  |
| Q1682 | B6J/AJ | F | 140 | Yes |  | Yes | Yes |
| R1341 | B6J/AJ | F | 140 | Yes |  | Yes | Yes |
| Q1754 | B6J/AJ | F | 141 | Yes |  | Yes | Yes |
| Q1680 | B6J/AJ | F | 144 | Yes |  | Yes | Yes |
| R1340 | B6J/AJ | F | 144 | Yes |  | x | x |
| Q1681 | B6J/AJ | F | 136 | Yes |  |  |  |
| R1865 | B6J/AJ | F | 139 | Yes |  |  |  |
| R1468 | B6J/AJ | F | 140 | Yes |  |  |  |
| R1496 | B6J/AJ | F | 142 | Yes |  |  |  |
| R1863 | B6J/AJ | F | 143 | Yes |  |  |  |
| Q1957 | B6J/AJ | F | 149 | Yes |  |  |  |
| R1604 | B6J/AJ | F | 141 | N.A. |  |  |  |
| R1916 | B6J/AJ | F | 146 | N.A. |  |  |  |
| R1866 | B6J/AJ | M | 137 | Yes | Yes |  |  |
| R1343 | B6J/AJ | M | 140 | Yes | Yes |  |  |
| Q1764 | B6J/AJ | M | 142 | Yes | Yes |  |  |
| R1344 | B6J/AJ | M | 143 | Yes | Yes |  |  |
| Q1961 | B6J/AJ | M | 140 | N.A. | Yes |  |  |
| R1342 | B6J/AJ | M | 141 | Yes |  | Yes | Yes |
| R1608 | B6J/AJ | M | 140 | N.A. |  | Yes | Yes |
| R1918 | B6J/AJ | M | 140 | N.A. |  | Yes | Yes |
| R1919 | B6J/AJ | M | 140 | N.A. |  | Yes | Yes |
| Q1960 | B6J/AJ | M | 145 | N.A. |  | Yes | Yes |

**Abbreviations:** Chr chromosome; F female; M male; N.A. not ascertained; x failed; WT wild type (Hdh^+/+^).
